# Impact of anti-inflammatory diets on cardiovascular disease risk factors: a systematic review and meta-analysis

**DOI:** 10.3389/fnut.2025.1549831

**Published:** 2025-03-20

**Authors:** Ruixue Jiang, Ting Wang, Kunlin Han, Peiqiang Peng, Gaoning Zhang, Hanyu Wang, Lijing Zhao, Hang Liang, Xuejiao Lv, Yanwei Du

**Affiliations:** ^1^Department of Respiratory and Critical Care Medicine, The Second Hospital of Jilin University, Changchun, Jilin Province, China; ^2^Department of Rehabilitation, School of Nursing, Jilin University, Changchun, Jilin Province, China

**Keywords:** anti-inflammatory diets, cardiovascular disease risk factors, blood pressure, lipids, hs-CRP, meta-analysis

## Abstract

**Introduction:**

Chronic inflammation, via multiple pathways, influences blood pressure and lipid profiles, serving as a significant risk factor for the onset of cardiovascular disease (CVD). Anti-inflammatory dietary patterns may ameliorate CVD risk factors through the modulation of inflammatory mediators and metabolic factors, potentially leading to improved cardiovascular outcomes. Current findings regarding the relationship between dietary habits and CVD risk factors, such as blood pressure and lipid levels, exhibit considerable variability. We performed a comprehensive systematic review and meta-analysis to explore the possible association between anti-inflammatory dietary patterns (such as the Mediterranean diet, DASH diet, Nordic diet, Ketogenic diet, and Vegetarian diet) and CVD risk factors.

**Methods:**

We conducted a comprehensive search across five databases: PubMed, Web of Science, Cochrane Library, Embase, and China National Knowledge Infrastructure (CNKI). Ultimately, we identified 18 eligible randomized controlled trials (including randomized crossover trials), which were subjected to meta-analysis utilizing RevMan 5 and Stata 18.

**Results:**

A comprehensive meta-analysis of these studies conducted based on random effects model indicated that, in comparison to an Omnivorous diet, interventions centered on anti-inflammatory diets were linked to significant reductions in Systolic Blood Pressure (SBP) (MD: −3.99, 95% CI: −6.01 to −1.97; *p* = 0.0001), Diastolic Blood Pressure (DBP) (MD: −1.81, 95% CI: −2.73 to −0.88; *p* = 0.0001), Low Density Lipoprotein Cholesterol (LDL-C) (SMD: −0.23, 95% CI: −0.39 to −0.07; *p* = 0.004), Total Cholesterol (TC) (SMD: −0.31, 95% CI: −0.43 to −0.18; *p* < 0.00001) and High-sensitivity C-reactive Protein (hs-CRP) (SMD: −0.16, 95% CI: −0.31 to −0.00; *p* = 0.04). No notable correlations were identified between High Density Lipoprotein Cholesterol (HDL-C) and Triglycerides (TG).

**Discussion:**

The findings indicate that anti-inflammatory diets may lower serum hs-CRP levels and positively influence the reduction of CVD risk factors, such as blood pressure and lipid profiles, thereby contributing to the prevention and progression of cardiovascular conditions. Most of the outcome indicators had low heterogeneity; sensitivity analyses were subsequently conducted on outcome measures demonstrating substantial heterogeneity, revealing that the findings remained consistent.

## 1 Introduction

Cardiovascular Disease (CVD) is a heterogeneous group of disorders affecting the heart and blood vessels, encompassing atherosclerosis (coronary, cerebrovascular, and peripheral artery diseases), structural/functional abnormalities (heart failure, arrhythmias, valvular/congenital defects), and microvascular dysfunction (1). These conditions are marked by inflammation, oxidative stress, cellular proliferation, hypertrophy, and potentially abnormal remodeling of the heart or blood vessels (2, 3). Recent statistics indicate that more than 500 million individuals globally are impacted by CVD, with 20.5 million fatalities linked to CVD in 2021, accounting for nearly one-third of total global mortality (4). Given the persistent increase in CVD incidence and mortality across nearly all nations worldwide, it is imperative to identify modifiable risk factors for CVD prevention.

Inflammation represents the body’s immune response to inflammatory triggers or cellular injury (5). Chronic tissue damage leads to the release of pro-inflammatory cytokines, which in turn triggers ongoing systemic inflammation (6), a potential pathological state that could significantly influence the development of CVD (7). Research has indicated that several inflammatory proteins may be linked to the risk of CVD (8). Specifically, hs-CRP has been endorsed by a consortium of specialists from the Centers for Disease Control and Prevention and the American Heart Association as the most reliable clinical assay for evaluating and forecasting the risk of CVD (9, 10). In atherosclerotic lesions, chronic inflammation is closely associated not only to their progression but also plays a role in every phase of the thrombosis process (11). Simultaneously, damage to the vascular endothelium, oxidative stress, and thrombosis could serve as potential mechanisms through which chronic inflammation influences the pathogenesis of atherosclerosis (12). Thrombosis is linked to a heightened risk of acute coronary incidents and subsequently contributes to cardiovascular conditions, including myocardial infarction (MI) and stroke (13). If inflammation continues, macrophages penetrate the compromised endothelial barrier and phagocytize abnormal cholesterol, leading to plaque formation. As endothelial injury exacerbates and lipid accumulation in the arteries progresses, sustained inflammatory stimuli can result in the gradual enlargement of atherosclerotic plaques (14). The disruption of the arterial wall and subsequent thrombus formation can result in obstructions in the cardiovascular system in patients, potentially precipitating coronary artery disease and a range of additional cardiac disorders (15). Simultaneously, inflammatory alterations may facilitate the recurrence of atrial fibrillation (AF) (16), and elevated levels of hs-CRP may heighten the risk of AF recurrence (17, 18).

Hypertension, the most prevalent cardiovascular disorder, is the primary risk factor for cardiovascular conditions, including myocardial infarction (MI) and stroke (19). Research indicates that hypertension triggers oxidative stress within the vascular wall, subsequently facilitating the progression of atherosclerosis (20). Hypertension may also induce left ventricular hypertrophy, which, over time, can advance to both diastolic and systolic heart failure (21). In recent years, the association between inflammation and hypertension has gained significant attention, with research indicating that inflammatory mediators, cellular components, and biomarkers are linked to the onset, progression, and outcomes of hypertension (22).

Blood lipids encompass the aggregate levels of neutral fats, including TG and cholesterol, as well as various lipids such as phospholipids, glycolipids, and sterols present in the plasma. Dyslipidemia serves as a critical risk factor for CVD. A notable correlation exists between lipid concentrations and the incidence of coronary artery disease (23). An accumulation of lipoproteins, particularly LDL-C, occurs in the subendothelial region, where they undergo oxidative modification. These modified lipoproteins are preferentially taken up by macrophages and monocytes, initiating the atherosclerotic process (24, 25). It is projected that around 4.3 million fatalities occur globally each year due to elevated levels of LDL-C, representing 7.7% of global mortality (26). Furthermore, elevated TG levels are associated with an increased risk of CVD. Consequently, to thoroughly evaluate the CVD risk linked to blood lipids, clinical guidelines frequently advocate for a complete lipid profile assessment. Research indicates that the excessive production of specific pro-inflammatory mediators can contribute to the onset of lipid metabolism disorders (27).

The elevated global mortality rate associated with cardiovascular disease (CVD) presents a pressing challenge that necessitates immediate attention. Numerous researchers are concentrating their efforts on the development of effective pharmacological interventions for CVD, alongside investigations into the various risk factors contributing to cardiovascular health. Research indicates that the most significant risk factors contributing to CVD mortality include hypertension at 10.8%, followed closely by low educational attainment at 10.5%, suboptimal dietary habits at 8.3%, tobacco consumption at 7.5%, and exposure to household air pollution at 6.1% (28). A significant proportion of CVD fatalities can be averted by addressing modifiable lifestyle risk factors. Among these, dietary habits represent a crucial yet frequently neglected risk factor for CVD. Anti-inflammatory diets were initially introduced by Dr. Barry Sears (29), encompassing dietary models that systemically modulate inflammatory pathways through synergistic nutrient interactions. Currently, several evidence-based anti-inflammatory dietary patterns are prominent in clinical research, including Mediterranean diet, Nordic diet, DASH diet, Ketogenic diet, and Vegan diet. The Mediterranean Diet is characterized by high consumption of extra-virgin olive oil (≥60 mL/day), fatty fish (≥2 servings/week), and polyphenol-rich plant foods (fruits, vegetables, and whole grains) (30). The DASH Diet, initially designed for blood pressure control, emphasizes sodium restriction (<2,300 mg/day) combined with potassium-rich foods (such as fruits, vegetables, whole grains, nuts and seeds) and low-fat dairy. The Nordic Diet features locally sourced components including berries (≥100 g/day), cruciferous vegetables, and rapeseed oil. The Vegan Diet relies on legume-based proteins and flaxseed (≥30 g/day) to optimize omega-3/6 ratios. The ketogenic diet operates on a distinct metabolic paradigm requiring strict carbohydrate restriction (≤50 g/day) and high fat intake (70–80% of calories), exerting anti-inflammatory effects primarily through *β*-hydroxybutyrate-mediated NLRP3 inflammasome suppression (31). Presently, the inflammatory potential of dietary patterns can be assessed using the Dietary Inflammatory Index (DII), a tool that quantifies the influence of diet on bodily inflammation by analyzing the balance between pro-inflammatory and anti-inflammatory dietary components (32). A positive DII score indicates a pro-inflammatory dietary pattern, whereas a negative DII score signifies an anti-inflammatory dietary pattern (33, 34).

The Mediterranean and Nordic dietary patterns notably prioritize the consumption of beneficial unsaturated fats, including olive oil and omega-3 fatty acids sourced from fish. Current research indicates that diets abundant in olive oil may suppress the nuclear factor-κB (NF-κB) signaling pathway, thereby diminishing the secretion of pro-inflammatory cytokines, including tumor necrosis factor-alpha (TNF-*α*) and interleukin-6 (IL-6) (35). An abundant intake of vegetables and fruits serves as a significant reservoir of antioxidants, which can counteract free radicals and mitigate inflammation resulting from oxidative stress (36). The consumption of dietary fiber is believed to confer anti-inflammatory effects by enhancing the synthesis of anti-inflammatory short-chain fatty acids and various metabolites derived from the gut (37, 38). The omega-3 polyunsaturated fatty acids found in fish exhibit potent anti-inflammatory properties, particularly by the modulation of eicosanoid and resolving production (39, 40). The Ketogenic diet can induce a metabolic state known as ketosis, leading to the production of ketone bodies, including beta-hydroxybutyrate (41). Ketone bodies exhibit anti-inflammatory properties and are capable of inhibiting the NF-κB signaling pathway, thereby diminishing the secretion of pro-inflammatory cytokines (42).

As demonstrated previously, sustained adherence to anti-inflammatory diets may lead to a decrease in systemic inflammation markers; however, the definitive effects on CVD risk factors, such as blood pressure and lipid profiles, remain not yet fully established. Our objective is to aggregate robust evidence derived from systematic reviews and meta-analyses to examine the effects of anti-inflammatory diets on cardiovascular risk determinants, including blood pressure (SBP and DBP), lipid profiles (HDL-C, LDL-C, TG, TC), and inflammatory markers (hs-CRP).

## 2 Methods

We conducted this systematic review and meta-analysis in accordance with the guidelines outlined in the Preferred Reporting Items for Systematic Reviews and Meta-Analyses (PRISMA) statement (43).

### 2.1 Search strategy

Two independent reviewers performed a systematic literature review utilizing the PubMed, Web of Science, Cochrane Library databases, Embase and China National Knowledge Infrastructure (CNKI), from 2015 to January 25, 2025. Conduct a search in the English and Chinese databases utilizing the title/abstract or MeSH terms. The search strategy incorporated the following terms: (dietary inflammatory index or inflammatory diet or anti-inflammatory diet or dietary score or Mediterranean diet or DASH diet or Vegan diet or Nordic diet or Ketogenic diet or Vegetarian diet or Plant-based diet) and (cardiovascular disease or coronary heart disease or ischemic heart disease or myocardial infarction or stroke or heart attack or hypertension or CVD or CHD or MI or IHD or BP) and (random or placebo or double-blind). Furthermore, the reference lists of all eligible reviews or meta-analyses were meticulously examined to uncover any pertinent studies. Titles and abstracts of the identified papers were evaluated to select potentially relevant studies, and the complete texts of these articles were scrutinized to ascertain whether they contained all the necessary information. Each of these procedures was carried out independently by two reviewers, with any disagreements addressed through consultation with a third reviewer.

### 2.2 Inclusion and exclusion criteria

Studies that met the following criteria were included: (i) Interventions consisted of dietary patterns that exhibited anti-inflammatory properties, including the Mediterranean Diet, DASH Diet, Nordic Diet, Ketogenic Diet, and Vegetarian Diet. Alternatively, these interventions may have focused on dietary patterns that prioritize a synergistic blend of various nutrients and non-nutrients, characterized by a well-rounded nutritional profile that incorporates a higher intake of anti-inflammatory foods such as fresh fruits and vegetables, whole grains, legumes, fish, nuts, and natural spices, while minimizing the consumption of pro-inflammatory foods high in sugar, salt, and unhealthy fats; (ii) reporting CVD risk factor indicators or levels of inflammatory proteins post-intervention; (iii) reporting post-intervention outcome indicators measures should be presented as means and standard deviations, or medians and interquartile ranges; (iv) the study type was randomized controlled trial (RCT) or randomized controlled crossover trial (RCCT). If two or more different anti-inflammatory dietary interventions were present in the included randomized controlled crossover trial, the different anti-inflammatory diets were statistically combined to form the intervention group. Omnivorous diets with pro-inflammatory properties at baseline or interventions as pro-inflammatory diets served as control groups; and (v) The publication year of the study fell within the past decade.

Exclusion criteria included: (i) studies that did not measure the inflammatory potential of the diet or where the intervention group did not follow an anti-inflammatory dietary pattern; (ii) studies that did not report indicators of CVD risk factors or inflammatory markers; (iii) studies involving duplicate populations; (iv) study types that are observational (including cohort and case–control studies), cross-sectional studies, reviews, conference abstracts, case reports, editorials, letters, and commentaries; and (v) studies were published a decade ago.

### 2.3 Data extraction

A standardized data extraction form was utilized to collect information from each eligible study. The following details were collected: (i) the name of the first author; (ii) year of publication; (iii) type of study (RCT/RCCT); (iv) country of origin; (v) number of participants at baseline; (vi) age of the study population at baseline; (vii) gender distribution of participants; (viii) duration of the intervention; (ix) study design; (x) health status of participants at baseline; and (xi) outcomes. Data were extracted by two investigators independently. Any disagreement in screening the articles was resolved by discussion between the two investigators. Consultation with a third investigator was performed if necessary.

The intervention in the study involved an anti-inflammatory dietary pattern, which could include Mediterranean diet, DASH diet, Nordic diet, Ketogenic diet, or Vegetarian diet. Alternatively, the intervention may have focused on a dietary approach that emphasizes a combination of various nutrients and non-nutrients, characterized by a well-balanced nutritional profile. This profile includes an increased intake of anti-inflammatory foods such as fresh fruits and vegetables, whole grains, legumes, fish, nuts, and natural spices, while reducing the consumption of pro-inflammatory foods high in sugar, salt, and fat. The control group adhered to an Omnivorous diet with pro-inflammatory characteristics. Consequently, the intervention group was classified as following an anti-inflammatory diet, whereas the control group was categorized as adhering to a pro-inflammatory diet.

### 2.4 Quality assessment

Two reviewers independently utilized the Cochrane Collaboration’s Review Manager 5.3 risk assessment tool to evaluate the quality of the RCTs included in the study. The instrument offers seven criteria to evaluate various forms of bias, including selection bias, implementation bias, attrition bias, measurement bias, reporting bias, and additional biases. These criteria encompass random sequence generation, allocation concealment, participant and personnel blinding, outcome assessment blinding, incomplete outcome data, selective reporting, and other potential biases. To evaluate bias, each item was categorized into one of three options: “low risk,” “unclear risk” and “high risk.” Discrepancies in quality evaluation between the two reviewers were resolved through deliberation with a third reviewer.

### 2.5 Statistical analysis

Given the methodological discrepancies observed across the studies, we employed a random-effects model for the quantitative analysis of the outcome indicators. The I^2^ statistic was utilized to evaluate the heterogeneity among the studies (44), representing the proportion of total variation attributable to heterogeneity rather than random variation. We performed subgroup analyses based on study characteristics (such as intervention duration, geographical location, health status) for outcome indicators exhibiting significant heterogeneity in order to explore potential sources of this variability. Sensitivity analyses were conducted by systematically excluding individual studies for outcome indicators exhibiting significant heterogeneity, in order to evaluate the robustness of the findings. The assessment of publication bias was carried out through a visual examination of funnel plots and Egger’s test. All analyses were conducted utilizing Review Manager (RevMan) version 5.3 (Nordic Cochrane Center, Cochrane Collaboration Network, Copenhagen, Denmark) alongside Stata 18.

## 3 Results

### 3.1 Study selection

**Figure 1:**
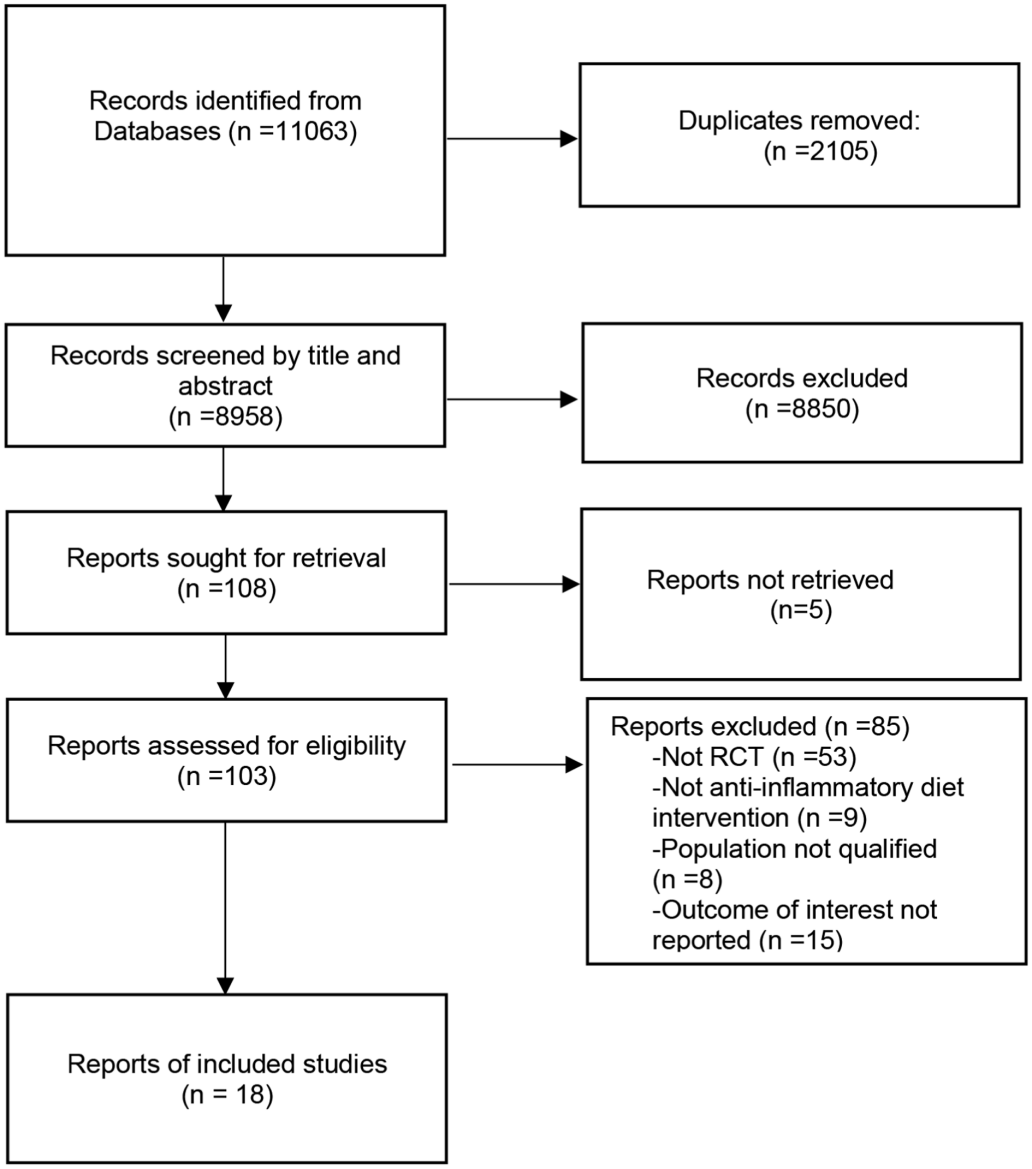
Flow chart of process of the selection.

The pertinence of the research was evaluated through the examination of titles, abstracts, and comprehensive texts. The entire procedure for identifying and selecting studies is illustrated in Figure 1. The search methodology yielded a total of 11,063 studies. Of these, 2,105 were eliminated due to duplication, while 8,850 were excluded following a review of titles and abstracts. Additionally, 5 studies were disregarded for failing to retrieve reports, and 85 were excluded after a comprehensive review of full texts, as they did not meet the specified criteria regarding study type, intervention measures, study population, and outcome indicators. Following the screening process, this meta-analysis concentrated on 18 eligible randomized controlled trials, including randomized crossover trials.

### 3.2 Study characteristics

**Figure 2:**
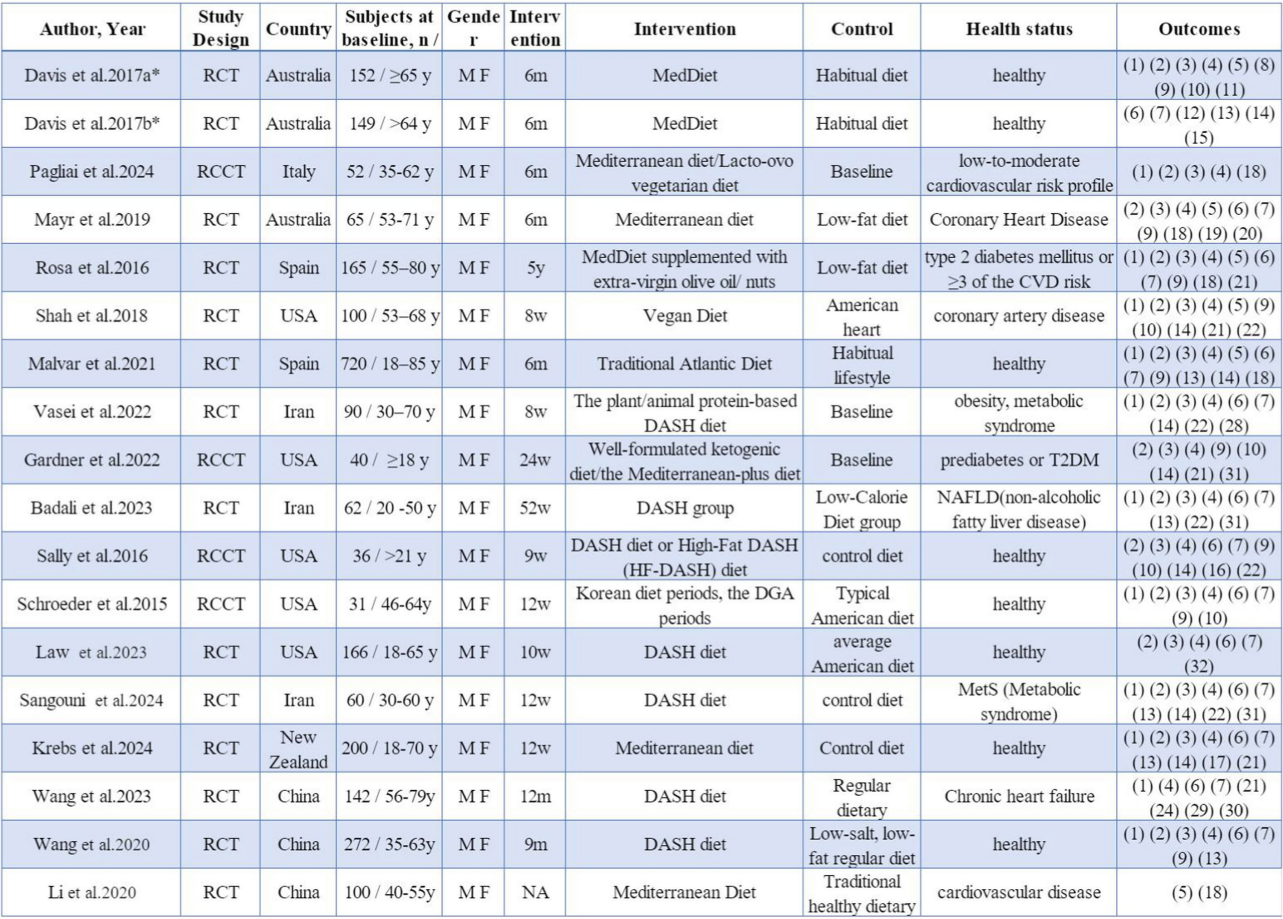
Study characteristics. * a and b indicate different studies with the same first author’s name. M, male; F, female; y, years; m, months; w, weeks; RCT, randomized controlled trial; RCCT, randomized controlled crossover trial. (1) Total cholesterol TC, (2) low density lipoprotein cholesterol LDL-C, (3) high density lipoprotein cholesterol HDL-C, (4) triglyceride TG, (5) high sensitivity C-reactive protein hs-CRP, (6) systolic blood pressure SBP, (7) diastolic blood pressure DBP, (8) cognitive function, (9) glucose GLU, (10) insulin, (11) F2-isoprostanes F2-IsoPs, (12) flow-mediated dilation FMD, (13) body mass index BMI, (14) body mass, (15) height; (16) the percentage of body fat BFP, (17) BIA fat mass, (18) cytokine CK, (19) adiponectin ADP, (20) malondialdehyde MDA, (21) glycosylated hemoglobin concentrations HbA1c, (22) waist circumference WC, (23) very low density lipoprotein VLDL, (24) quality of life, (25) comprehensive white blood cell-related biomarkers, (26) bioelectrical impedance analysis fat-free mass BIA FFM, (27) urine F2-isoprostane/creatinine ratio, (28) insulin resistance IR, (29) major adverse cardiovascular and cerebrovascular event MACCE, (30) left ventricular ejection fraction LVEF, (31) comprehensive liver function indicators, (32) lipoproteins LP, (33) hip circumference.

The studies included a total of 2,602 participants at baseline, with ages spanning from 18 to 85 years (Figure 2). All research indicated impacts on both sexes. Out of the 18 studies, 14 (45–58) were RCTs, while 4 (59–62) were RCCTs. A total of 9 studies featured intervention periods of 6 months or longer, while 8 studies had durations shorter than 6 months. These 18 studies were conducted across various regions, comprising Europe (3), North America (5), Oceania (4), and Asia (6).

### 3.3 Quality assessment

All studies included in the analysis were evaluated for potential bias utilizing the Cochrane Collaboration’s assessment tool, with the specifics of the quality evaluation illustrated in Supplementary Table 1. One study failed to disclose the methodology of randomization, six studies lacked details on allocation concealment, ten studies did not clarify the blinding of participants or researchers, six studies did not specify the blinding of outcome assessment, one study inadequately reported attrition and dropout rates, and two studies exhibited reporting bias.

### 3.4 Meta-analysis results

#### 3.4.1 Association of anti-inflammatory diets with blood pressure

**Figure 3:**
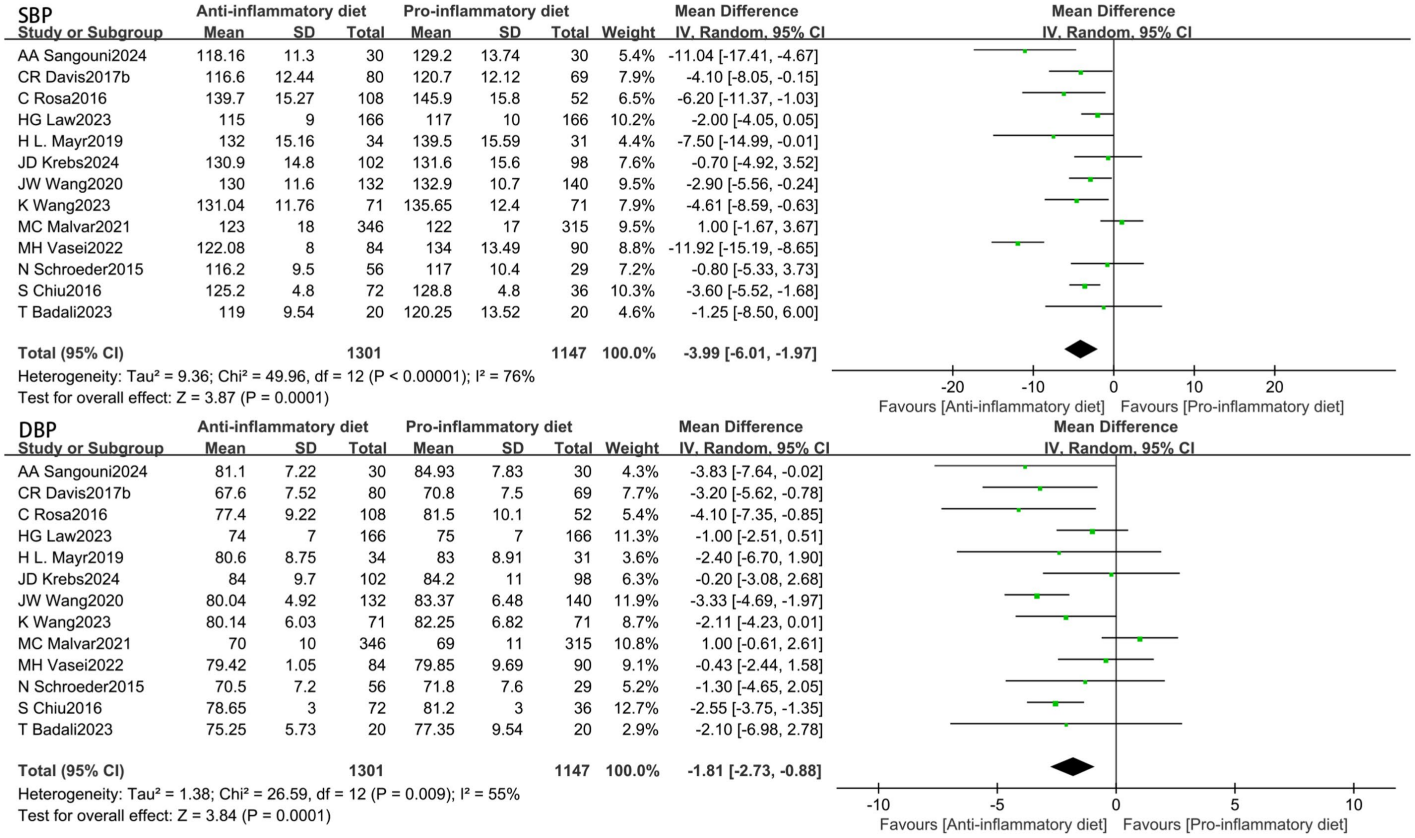
Random-effects meta-analysis and forest plot of the association between anti-inflammatory diets and blood pressure.

The aggregated findings from thirteen studies demonstrated that participants adhering to anti-inflammatory diets exhibited reduced blood pressure levels in comparison to those in the control group following the intervention. The substantial heterogeneity (I^2^ = 76%, *p* < 0.00001) indicated that the SBP was significantly lower in the anti-inflammatory diets intervention group when compared to the control group (MD: −3.99, 95% CI: −6.01 to −1.97, *p* = 0.0001) (Figure 3). Additionally, the Egger’s test was conducted, indicating the absence of publication bias (Supplementary Tables 2, 3). Conducting a sensitivity analysis by systematically excluding individual studies demonstrated that the study by Vasei was the primary source of heterogeneity. Its removal resulted in a reduction of heterogeneity to I^2^ = 49% (*p* = 0.03), while the effect size remained largely stable (MD: −2.96, 95% CI: −4.44 to −1.49, *p* < 0.0001). In the presence of moderate heterogeneity (I^2^ = 55%, *p* = 0.009), the anti-inflammatory diets group exhibited a significant reduction in DBP compared to the control group (MD: −1.81, 95% CI: −2.73 to −0.88, *p* = 0.0001) (Figure 3). A visual assessment of the funnel plot revealed no evidence of publication bias (Supplementary Tables 2, 3). When the result from Malar was excluded, heterogeneity decreased to I^2^ = 17% (*p* = 0.28), while the effect size remained largely consistent (MD: −2.17, 95% CI: −2.87 to −1.47, *p* < 0.00001).

#### 3.4.2 Association of anti-inflammatory diets with lipids

**Figure 4:**
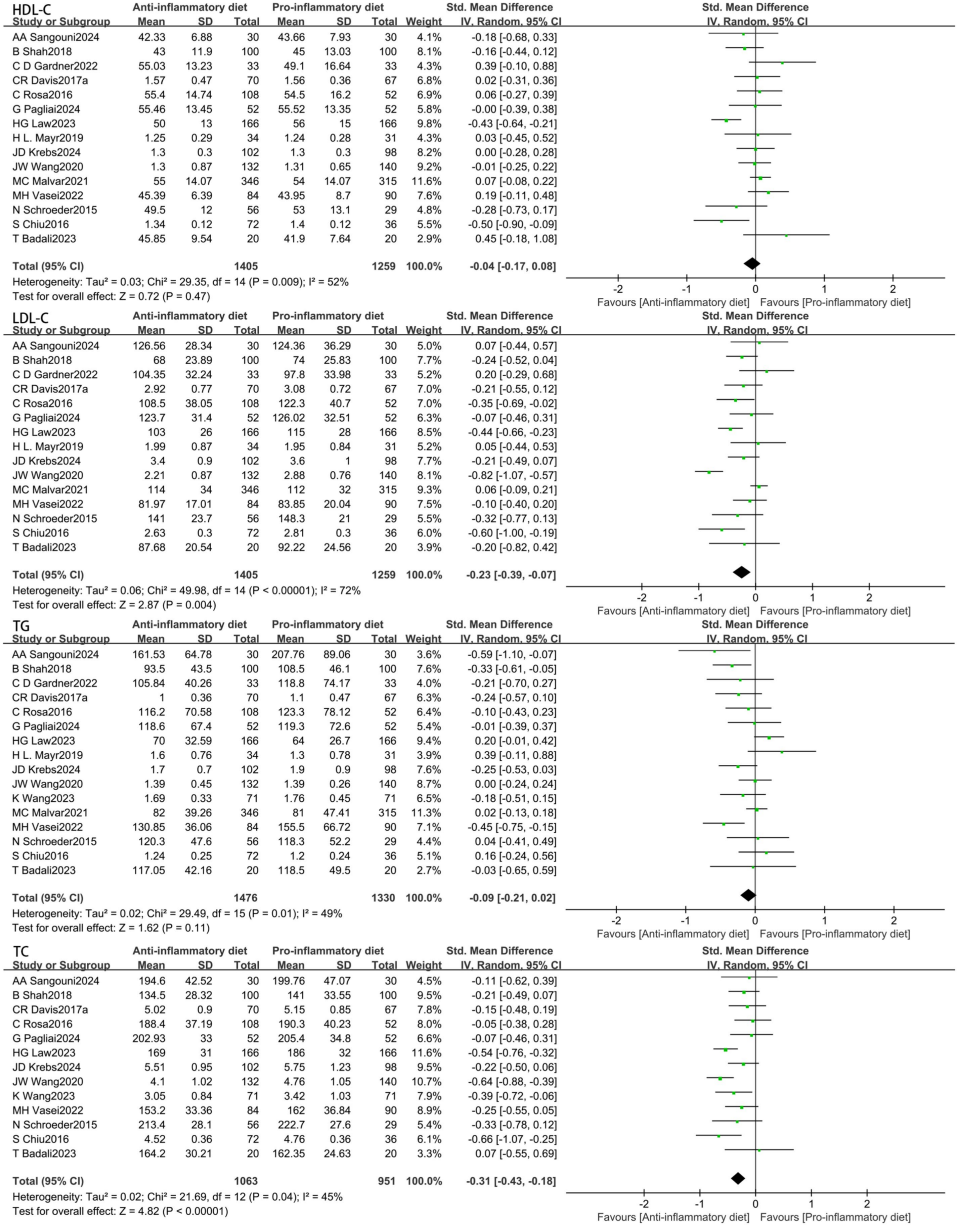
Random-effects meta-analysis and forest plot of the association between anti-inflammatory diets and lipids.

In these investigations, 16 articles analyzed the effects of an anti-inflammatory diet on TG, 15 studies evaluated the impact of an anti-inflammatory diet on HDL-C, LDL-C, while 13 studies focused on TC. No notable correlation was identified between the anti-inflammatory diet cohort and HDL-C levels compared with the control group (SMD: -0.04, 95% CI: −0.17 to 0.08, *p* = 0.47) (Figure 4). Nevertheless, moderate heterogeneity was detected across the studies (I^2^ = 52%, *p* = 0.009). The visual assessment of funnel plots, along with the results from Egger’s test, suggested that there is no evidence of publication bias (Supplementary Tables 2, 3). Sensitivity analysis, which involved the exclusion of certain studies, identified Law’s result as the primary contributor to heterogeneity. By omitting this study, heterogeneity was reduced to I^2^ = 18% (*p* = 0.26), while the effect size remained largely consistent (SMD = 0.00, 95% CI: −0.09 to 0.10, *p* = 0.96).

With moderate heterogeneity (I^2^ = 72%, *p* < 0.00001), the anti-inflammatory diets group lowered LDL-C compared with the control group (SMD: -0.23, 95% CI: −0.39 to −0.07, *p* = 0.004) (Figure 4). The visual examination of funnel plots and the Egger’s test indicated the absence of publication bias (Supplementary Tables 2, 3). Sensitivity analyses showed that Wang’s (57) results were the largest contributor to heterogeneity, and excluding them reduced heterogeneity to I^2^ = 48% (*p* = 0.02), with essentially unchanged effect sizes (SMD: -0.18, 95% CI: −0.30 to −0.06, *p* = 0.004).

Additionally, the TC levels were also significantly lower in the anti-inflammatory diets group compared to the control group (SMD: -0.31, 95% CI: −0.43 to −0.18, *p* < 0.00001) (Figure 4), with low heterogeneity observed (I^2^ = 45%, *p* = 0.04). Under less heterogeneity (I^2^ = 49%, *p* = 0.01), no statistically significant relationship was observed between the anti-inflammatory diets cohort and TG levels when compared to the control group (SMD: -0.09, 95% CI: −0.21 to 0.02, *p* = 0.11) (Figure 4).

#### 3.4.3 Association of anti-inflammatory diets with hs-CRP

**Figure 5:**
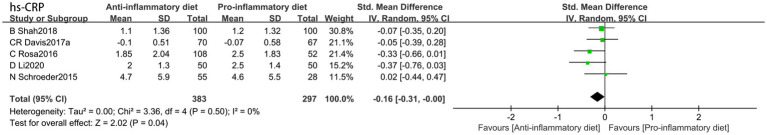
Random-effects meta-analysis and forest plot of the association between pro-inflammatory diet and high-sensitivity c-reactive protein.

In comparison to the control group, the anti-inflammatory dietary intervention demonstrated a significant reduction in hs-CRP (SMD: −0.16, 95% CI: −0.31 to −0.00, *p* = 0.04) (Figure 5), exhibiting no heterogeneity (I^2^ = 0%, *p* = 0.50).

### 3.5 Risk of publication bias

The visual examination of funnel plots and the application of Egger’s test indicated an absence of publication bias across all metrics in this analysis (Supplementary Tables 2, 3).

### 3.6 Subgroup analysis

**Figure 6:**
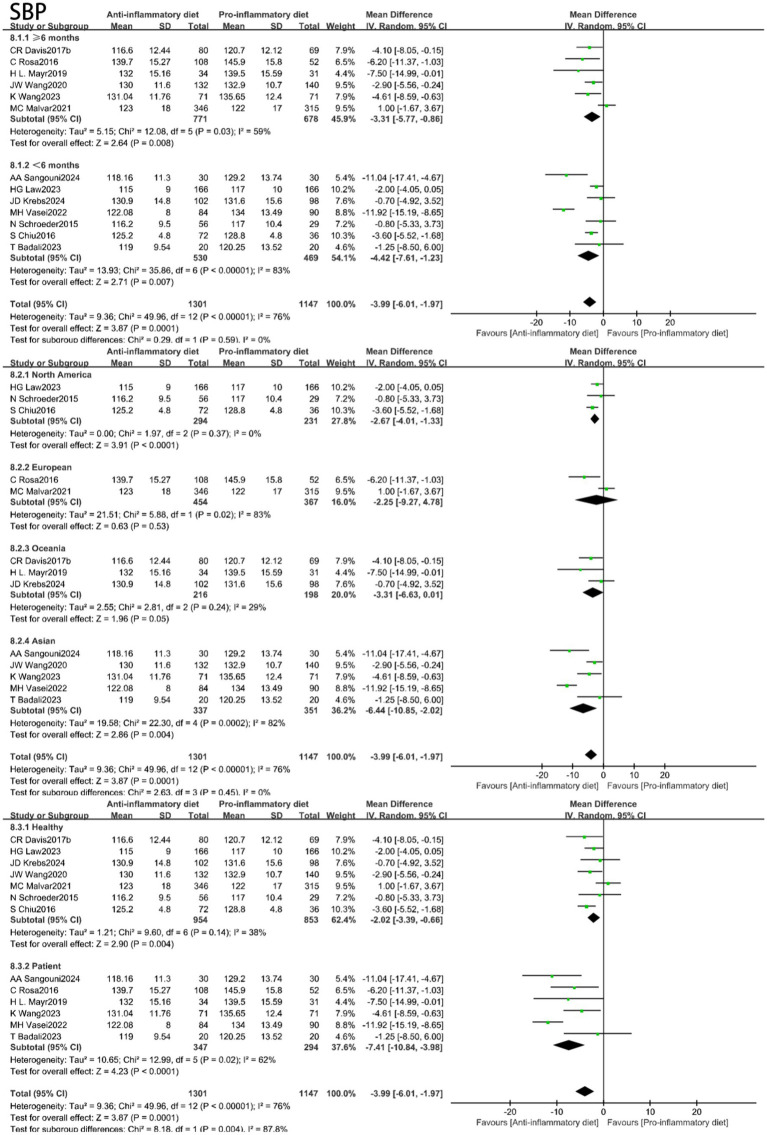
Subgroup analyses of systolic blood pressure (SBP).

**Figure 7:**
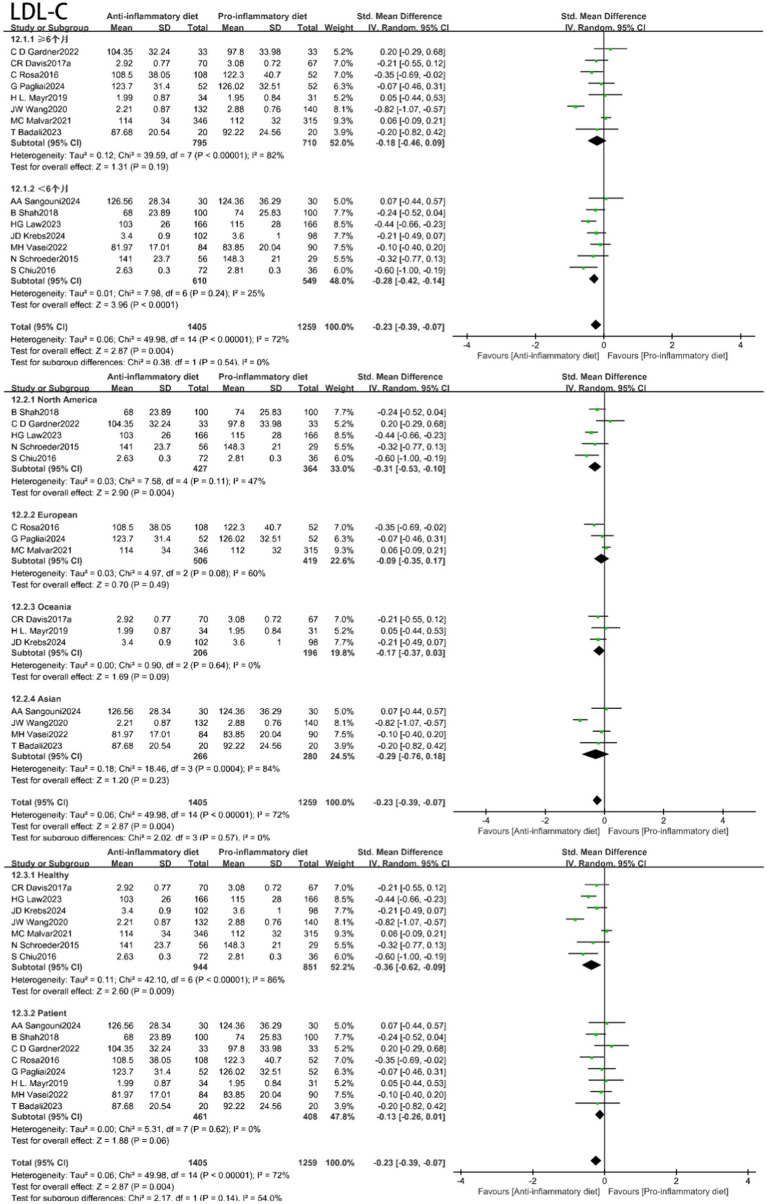
Subgroup analyses of diastolic low density lipoprotein cholesterol (LDL-C).

**Figure 8:**
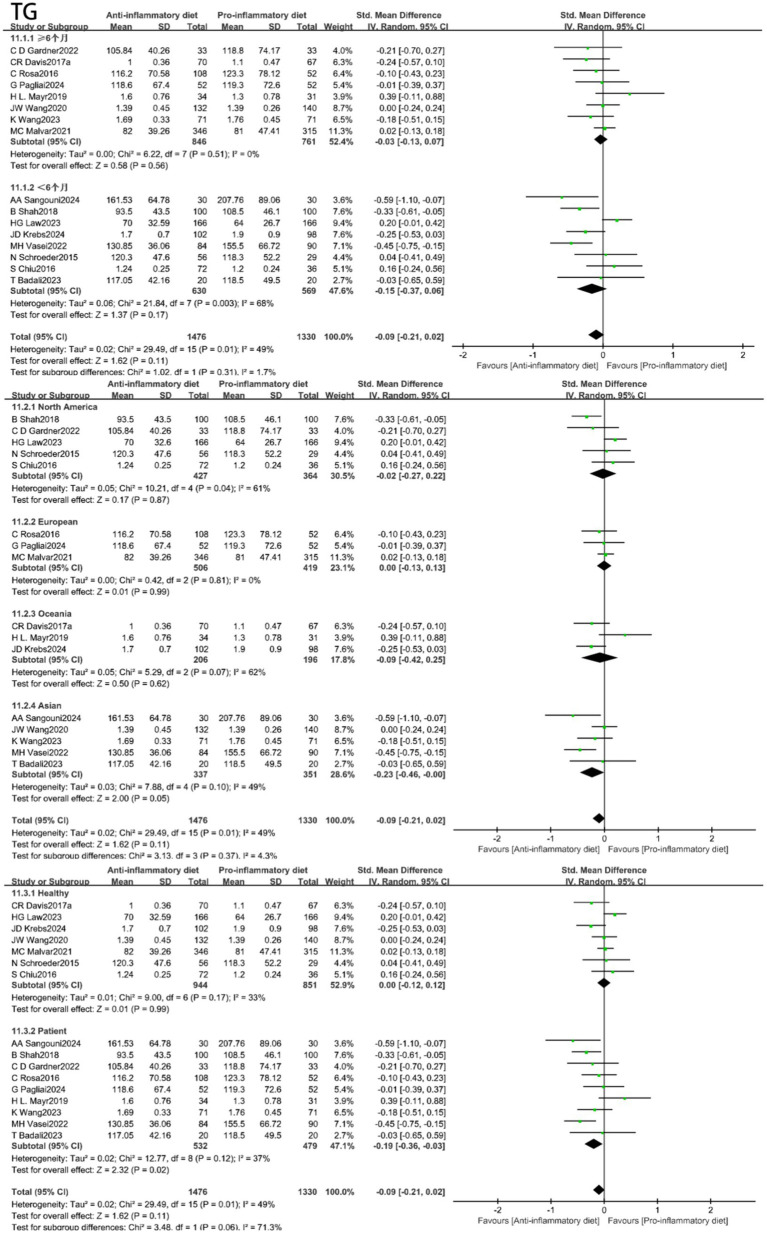
Subgroup analyses of triglyceride (TG).

In light of the substantial heterogeneity identified across the studies, we conducted subgroup analyses for SBP, DBP, HDL-C, LDL-C and TG by stratifying based on intervention duration, geographical location, and health status. The results are depicted in Figures 6–8 and Supplementary Tables 3, 4. For SBP, subgroup analysis revealed model sensitivity to intervention duration, geographical region, and health status, suggesting that the observed heterogeneity may primarily stem from regional disparities. For DBP, the subgroup analysis indicated that the effect model was similarly impacted by intervention duration, geographical region, and health status; thus, the heterogeneity observed in the studies may be attributed to these intervention duration and health condition. Regarding HDL-C, the subgroup analysis revealed that the effect model was likewise influenced by intervention duration, geographical region, and health status; consequently, the heterogeneity in the studies could be associated with the duration of the intervention, geographical location, and health status. In the case of LDL-C, the subgroup analyses indicated that the effect model was affected by factors such as intervention duration, geographical region, and health status; the heterogeneity observed in the studies may stem from the influences of region and health status. Subgroup analyses of triglycerides indicated that the effect model was affected by the duration of the intervention, geographical region, and health status. Furthermore, the anti-inflammatory diets demonstrated efficacy in lowering triglyceride levels among individuals with pre-existing conditions at baseline.

## 4 Discussion

This systematic review and meta-analysis encompassing 18 RCTs, with an initial cohort of 2,602 participants, indicates that individuals adhering to anti-inflammatory dietary interventions exhibited significantly lower levels of blood pressure (systolic and diastolic), LDL-C, TC, and hs-CRP compared to control groups consuming omnivorous diets. These findings suggest that strategic dietary modifications limiting pro-inflammatory foods (e.g., red meat) while enhancing the consumption of anti-inflammatory elements such as fruits and vegetables may effectively reduce systemic inflammation and CVD risk factors.

Our results align with recent evaluations concerning the DII and its relationship with CVD risk. This suggests that adherence to healthier dietary patterns, specifically anti-inflammatory diets, is associated with reduced incidence of cardiovascular events in both RCTs and observational studies (63–65). The cardioprotective mechanisms of anti-inflammatory dietary patterns may be mediated through the reduction of serum hs-CRP concentrations. As a key inflammatory biomarker, hs-CRP functions as an acute-phase reactant that impairs endothelial progenitor cell differentiation, viability, and functionality by downregulating endothelial nitric oxide synthase (eNOS) expression. This cascade promotes inflammatory cell infiltration and elevates oxidative stress, ultimately accelerating atherosclerosis progression via oxidative stress-mediated pathways (66, 67). Hs-CRP induces plasminogen activator inhibitor-1 (PAI-1) synthesis in endothelial cells via upregulation of endothelin-1 and IL-6 expression (68). Elevated PAI-1 promotes vascular thrombosis by modulating thrombotic factors (69). Anti-inflammatory dietary patterns demonstrate the capacity to reduce serum hs-CRP levels while improving endothelial function. Endothelial cells constitutively release vasoactive mediators such as prostacyclin (PGI2) and nitric oxide (NO), critical regulators of vascular tone and blood pressure homeostasis (70). These interventions reduce lipid accumulation and inflammatory cell adhesion (70), improving blood lipid profiles. This dietary approach attenuates chronic inflammation through dual mechanisms: oxidative stress reduction and insulin sensitivity improvement. Dietary antioxidants demonstrate anti-inflammatory effects through free radical scavenging and oxidative stress reduction (71). This dietary pattern attenuates chronic inflammation via dual pathways: enhancing insulin sensitivity while mitigating insulin resistance (72).

Furthermore, subgroup analysis findings suggested that, regarding blood pressure and lipid indicators, the regional distribution and baseline health status of participants during the intervention were associated with increased heterogeneity. These findings highlight how geographic distribution, cultural contexts, and baseline health status may modify the associations between anti-inflammatory diets and CVD risk factors. Consequently, it is crucial to investigate both feasibility and implementation challenges of anti-inflammatory dietary patterns considering geographical diversity and cultural traditions. Originating primarily in Mediterranean and Nordic regions, these dietary patterns face documented implementation barriers in other geographical settings, leading to substantially low adherence rates in many populations (73, 74). The traditional anti-inflammatory dietary patterns may not align with the culinary and dietary practices prevalent in specific regions. Altering entrenched dietary habits constitutes a significant challenge requiring gradual implementation. Enabling individuals to adjust their daily nutritional needs in accordance with their dietary preferences, while utilizing local food conversion charts that adhere to the tenets of the anti-inflammatory dietary framework, could enhance adherence to some degree (75). Successful adoption of an anti-inflammatory diet necessitates social support. Government initiatives can play a pivotal role in educating the populace about this diet by promoting the use of local agricultural products and aligning with seasonal availability. For instance, individuals can be encouraged to enhance their consumption of whole grains while minimizing the intake of highly processed staple foods; to enjoy their preferred vegetables while increasing the intake of cost-effective fruits; to elevate their consumption of fish, shrimp, and shellfish while decreasing red meat intake; to utilize appropriate amounts of monounsaturated fats and oils (such as olive oil and tea oil) in cooking, while also reducing dietary sugar; and to cultivate the habit of incorporating nuts into their diet (76).

The findings suggest that the anti-inflammatory diets are poised to be a pivotal strategy in mitigating the global burden of CVD, with significant practical implications for dietitians, healthcare professionals, and the broader populace. Initially, it furnishes dietitians with a more robust scientific foundation for incorporating anti-inflammatory diets into tailored dietary recommendations for individuals at risk of CVD. Concurrently, dietitians can leverage the DII to evaluate patients’ dietary patterns and adapt anti-inflammatory dietary regimens dynamically, in conjunction with metabolic markers (e.g., hs-CRP and lipid profiles), on an individual basis (33). For healthcare providers, anti-inflammatory diets may serve as a nonpharmacological intervention for both primary and secondary prevention of CVD, potentially synergizing with pharmacological treatments (77). Furthermore, the results support the integration of inflammatory markers (e.g., hs-CRP) into CVD risk assessment models and their application in monitoring the biological effects of anti-inflammatory dietary interventions. Sustained adherence to an anti-inflammatory diet may prove effective in reducing healthcare expenditures within the general population, while simultaneously retarding the progression of atherosclerosis and enhancing vascular endothelial function, thereby promoting healthy aging.

Our meta-analysis offers several significant advantages. Firstly, unlike previous meta-analyses that primarily included cross-sectional studies, prospective cohort studies, and case–control studies, our analysis distinctly incorporates RCTs (including RCCTs), thereby bolstering the evidential robustness of the original studies considered. Secondly, we implemented a comprehensive search strategy across various databases, covering both English and non-English literature, which substantially reduces the risk of overlooking eligible studies. Finally, by concentrating on research conducted in the past decade, we ensured the data’s relevance and timeliness, thereby enhancing the overall quality of the meta-analysis. At the same time, this meta-analysis has some limitations. Firstly, several of the studies included did not employ a quantitative scoring system based on the DII and instead relied on prior definitions of anti-inflammatory dietary patterns to assess whether the intervention constituted an anti-inflammatory diet. Secondly, certain studies exhibit a discernible implementation bias, given the inherent challenges in blinding participants to dietary interventions. Consequently, this may exert an influence on the observed intervention outcomes. Thirdly, the results from the subgroup analyses indicated that the impact of anti-inflammatory dietary interventions on HDL-C may begin to manifest after approximately 6 months. However, due to significant variations in intervention durations across the included studies, it was not feasible to further stratify the intervention durations into subgroups for analysis, thereby precluding the determination of the optimal duration for which an anti-inflammatory diet could enhance HDL-C levels.

The findings of this meta-analysis indicate that anti-inflammatory dietary patterns may contribute to the reduction of inflammation markers and the enhancement of CVD risk factors, thereby offering significant implications for the prevention and management of CVD. However, these findings should be interpreted cautiously, given the limited number of included studies, with high-sensitivity C-reactive protein (hs-CRP) data available from only five studies. Furthermore, while some studies included comprehensive food lists, others did not. Consequently, we advocate for future research to enhance sample sizes, refine study methodologies, and furnish more detailed dietary inventories.

## 5 Conclusion

In conclusion, the findings of this meta-analysis demonstrate that an anti-inflammatory dietary pattern is associated with reduced serum hs-CRP concentrations, significant reductions in blood pressure, and improvements in lipid profiles. These results suggest that adopting an anti-inflammatory diet may mitigate CVD risk. However, given the observed heterogeneity and limitations discussed earlier, additional high-quality, large-scale RCTs with rigorous methodology are required to confirm these findings.

## Data Availability

The original contributions presented in the study are included in the article/Supplementary material, further inquiries can be directed to the corresponding authors.
