# Supplementary material for: Impact of anti-inflammatory diets on cardiovascular disease risk factors: a systematic review and meta-analysis
Source: Front Nutr. 2025 Mar 20;12:1549831. doi: 10.3389/fnut.2025.1549831 (PMC11965126; doi:10.3389/fnut.2025.1549831)
Supplement: Supplementary file 1 [file Data_Sheet_1.docx]

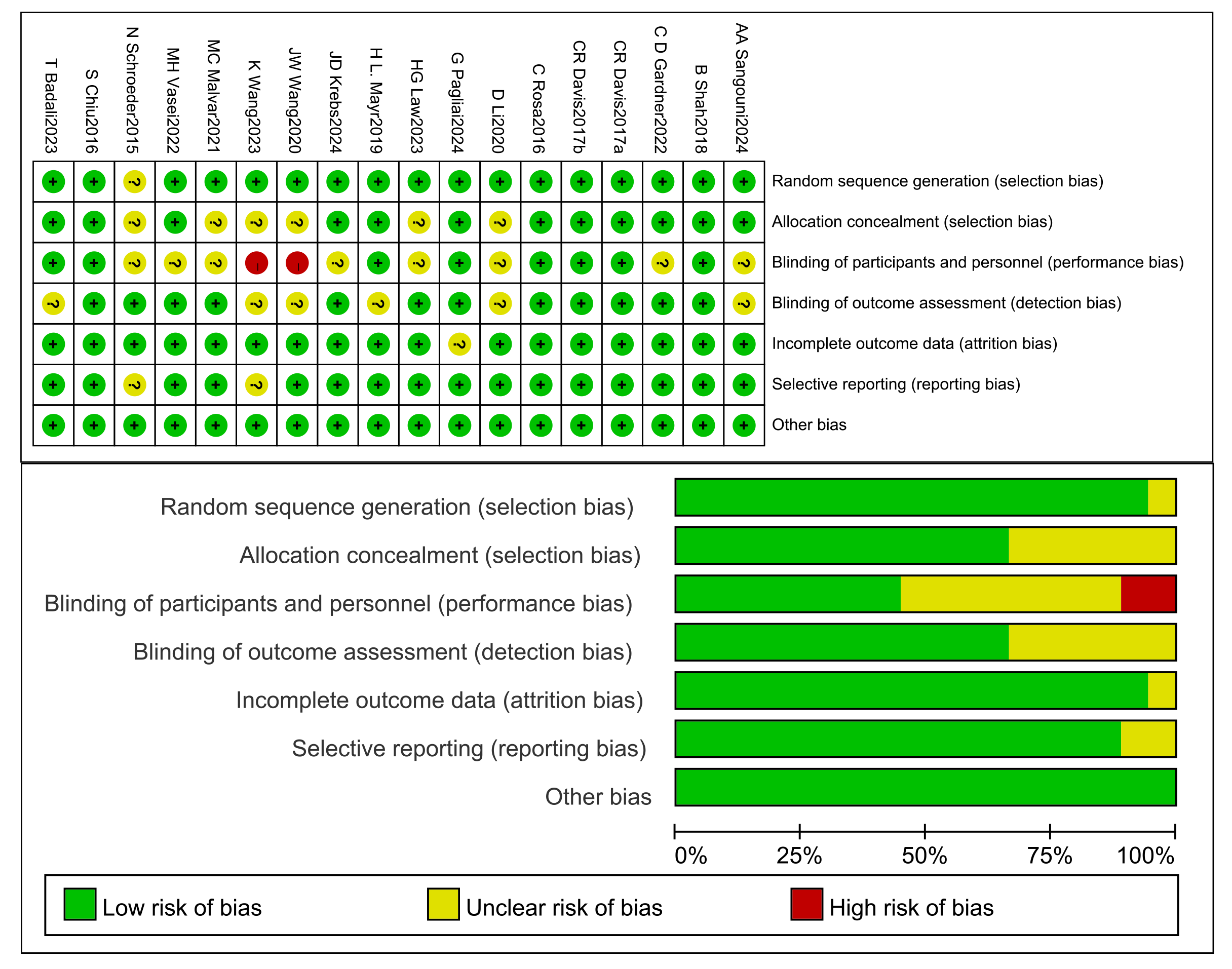


Supplementary table 1 Risk of bias graph


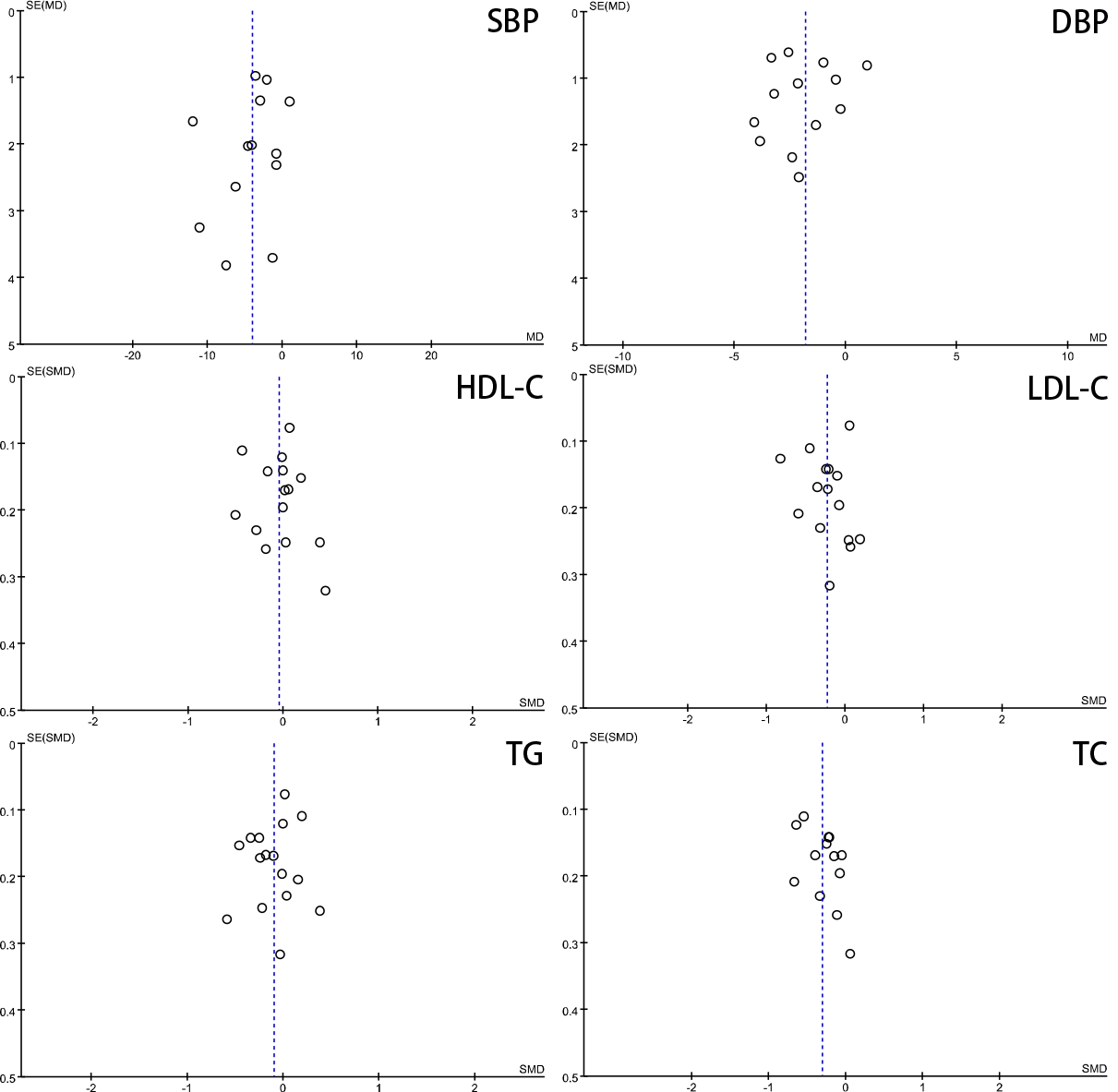


Supplementary table 2 Funnel plot


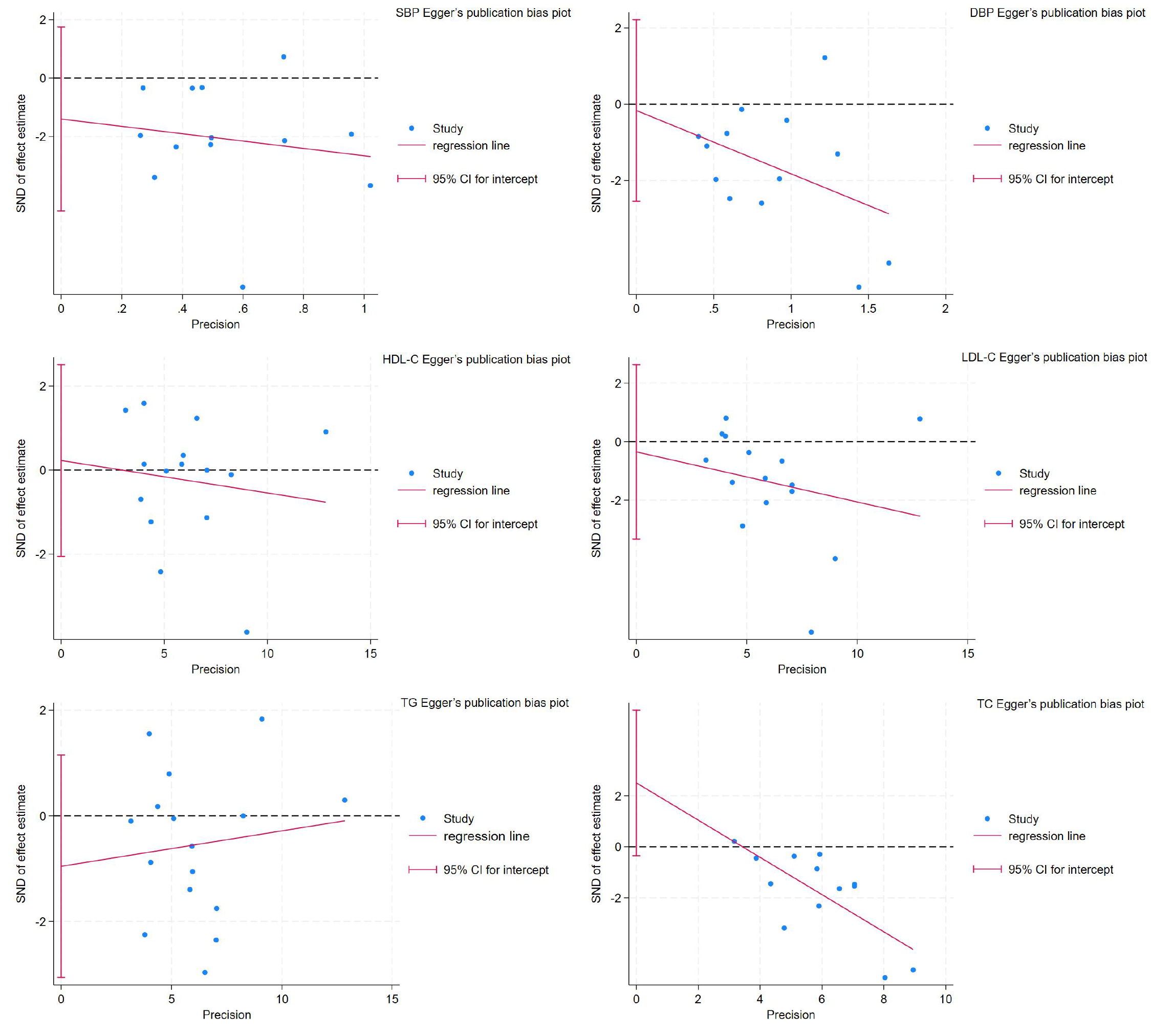


Supplementary table 3 Egger's test publication bias plot


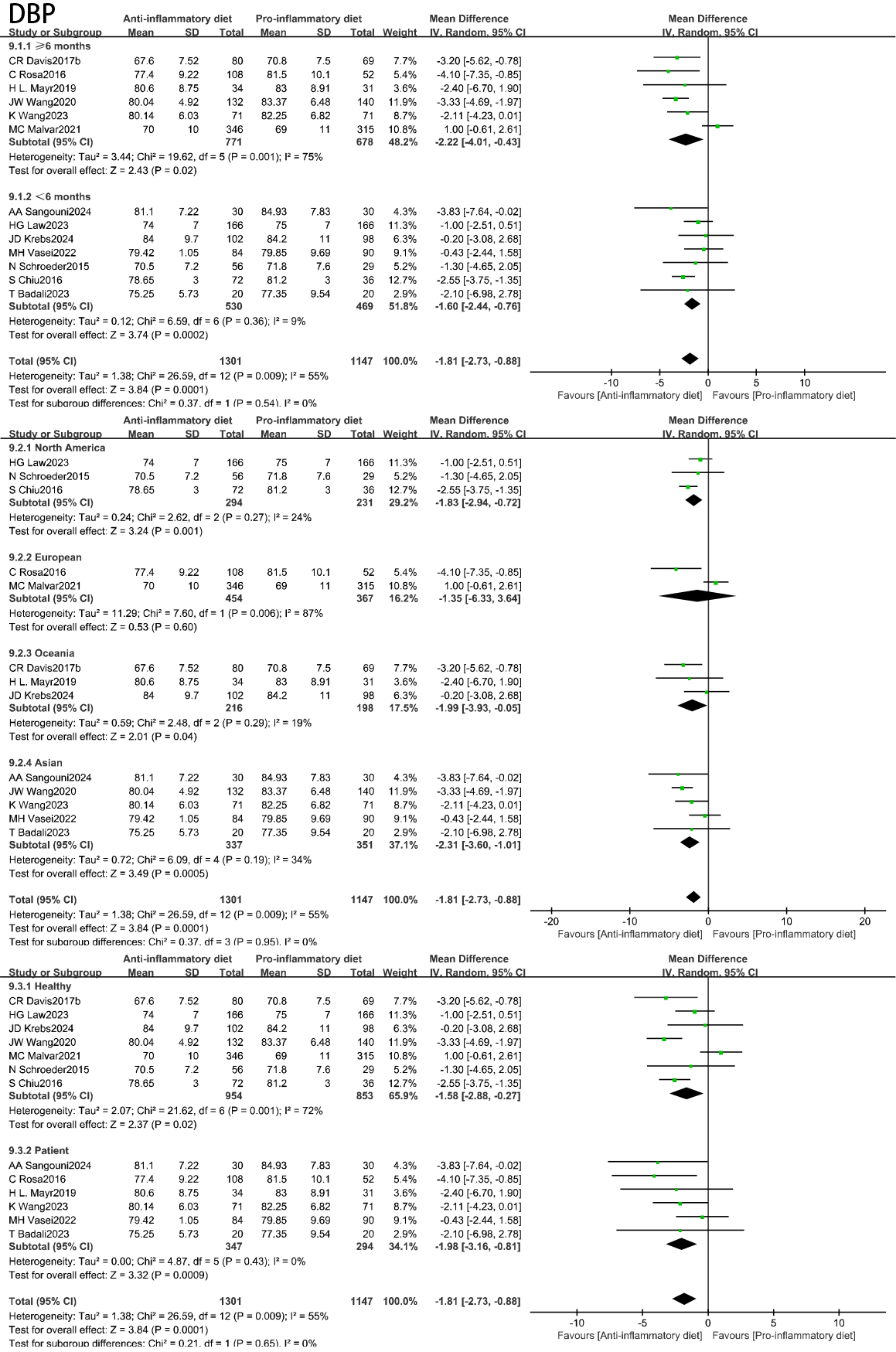


Supplementary table 4 Subgroup analyses of diastolic blood pressure (DBP)


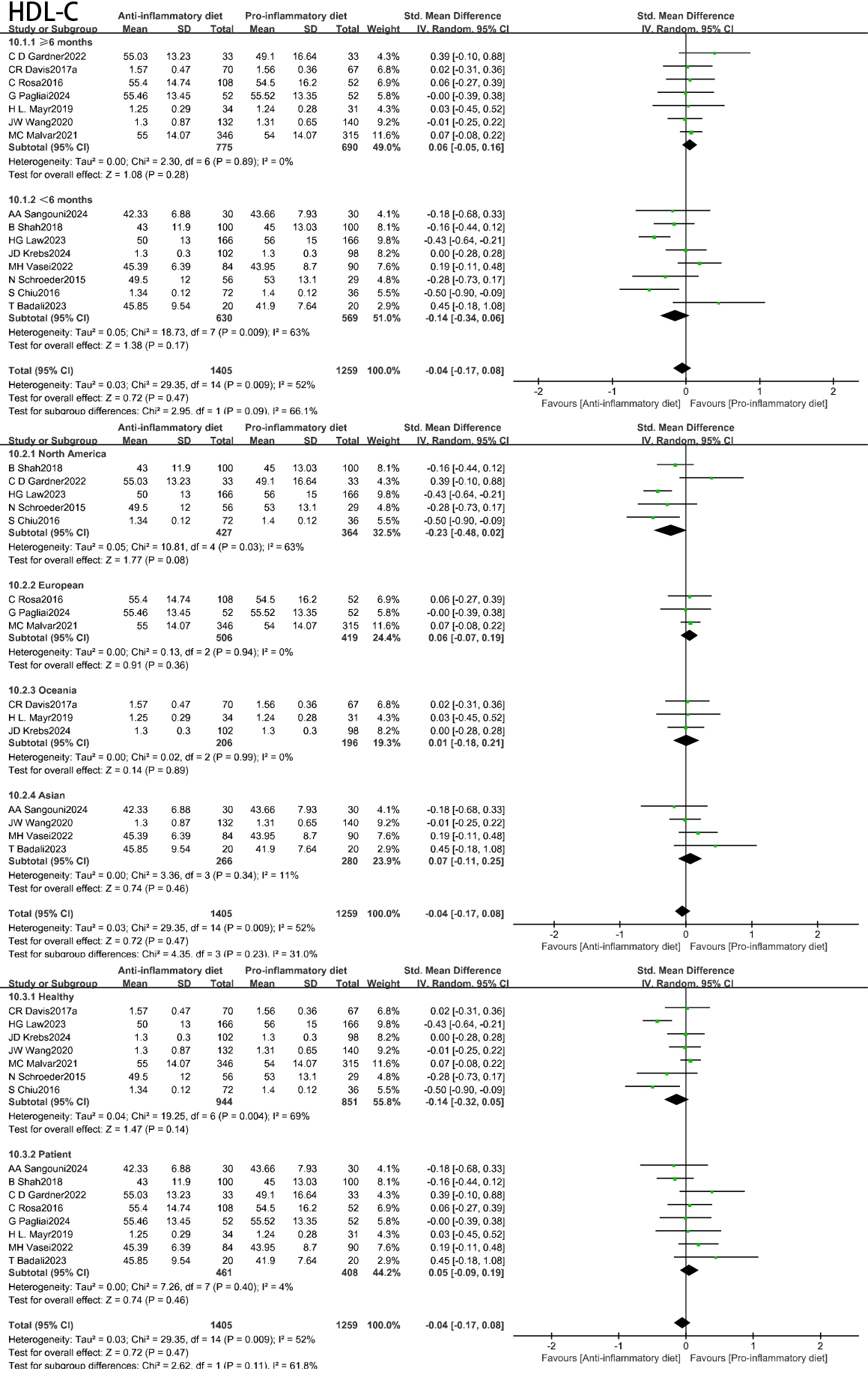


Supplementary table 5 Subgroup analyses of high-density lipoprotein cholesterol (HDL-C)

| Database name | Search strategies: key words and how these were combined in the search | Laster update | Number of studies identified |
| --- | --- | --- | --- |
| PubMed | #1"Cardiovascular Diseases"[MeSH Terms] OR "cardiovascular disease"[Title/Abstract] OR "disease cardiovascular"[Title/Abstract] OR "cardiac events"[Title/Abstract] OR "cardiac event"[Title/Abstract] OR "event cardiac"[Title/Abstract] OR "adverse cardiac event*"[Title/Abstract] OR "cardiac event* adverse"[Title/Abstract] OR "major adverse cardiac events"[Title/Abstract] OR "Coronary Disease"[MeSH Terms] OR "Myocardial Ischemia"[MeSH Terms] OR "Myocardial Infarction"[MeSH Terms] OR "Stroke"[MeSH Terms] OR "Hypertension"[MeSH Terms] OR "heart attack"[Title/Abstract] OR "CVD"[Title/Abstract] OR "CHD"[Title/Abstract] OR "MI"[Title/Abstract] OR "HP"[Title/Abstract] OR "IHD"[Title/Abstract] 3001845  #2"dietary inflammatory index"[Title/Abstract] OR "DII"[Title/Abstract] OR "inflammatory diet"[Title/Abstract] OR "anti inflammatory diet"[Title/Abstract] OR "dietary inflammation index"[Title/Abstract] OR "anti inflammation diet"[Title/Abstract] OR "inflammation diet"[Title/Abstract] OR "dietary score"[Title/Abstract] OR "diet scores"[Title/Abstract] OR "Mediterranean diet"[Title/Abstract] OR "DASH diet"[Title/Abstract] OR "vegan diet"[Title/Abstract] OR "Nordic diet"[Title/Abstract] OR "ketogenic diet"[Title/Abstract] OR "vegetarian diet"[Title/Abstract] OR "plant-based diet"[Title/Abstract] 23602  #3"random" OR "placebo" OR "double-blind"1713182  #4 #1AND#2AND#31039 | ^25th^  Jan.  2025 | 1039 |
| Web of science | #1TS=(Cardiovascular Disease) OR TS=(Disease, Cardiovascular) OR TS=(Cardiac Events) OR TS=(Cardiac Event) OR TS=(Event, Cardiac) OR TS=(Adverse Cardiac Event) OR TS=(Adverse Cardiac Events) OR TS=(Cardiac Event, Adverse) OR TS=(Cardiac Events, Adverse) OR TS=(Major Adverse Cardiac Events) OR TS=(CVD) OR TS=(coronary heart disease) OR TS=(CHD) OR TS=(ischemic heart disease) OR TS=(Myocardial Ischemia) OR TS=(IHD) OR TS=(myocardial infarction) OR TS=(stroke) OR TS=(apoplexy) OR TS=(heart attack) OR TS=(hypertension) OR TS=(high blood pressure) OR TS=(hypertensive) OR TS=(cardiovascular system) 5211321  #2 TS=(dietary inflammatory index) OR TS=(DII) OR TS=(inflammatory diet) OR TS=(anti-inflammatory diet) OR TS=(dietary inflammation index) OR TS=(anti-inflammation diet) OR TS=(inflammation diet) OR TS=(dietary score) OR TS=(diet scores) OR TS=(Mediterranean diet ) OR TS=(DASH diet) OR TS=(vegan diet ) OR TS=(Nordic diet ) OR TS=(ketogenic diet) OR TS=(vegetarian diet) OR TS=(plant-based diet) 288214  #3 TS=(random) OR TS=(placebo) OR TS= (double-blind) 2036533  #4 #1 AND #2 AND #3 5240 | ^25th^  Jan.  2025 | 5240 |
| The Cochrane library | #1MeSH descriptor: [Cardiovascular Diseases] explode all trees 159850  #2 MeSH descriptor: [Coronary Disease] explode all trees 19277  #3MeSH descriptor: [Myocardial Ischemia]  explode all trees 39343  #4MeSH descriptor: [Myocardial Infarction] explode all trees 15921  #5MeSH descriptor: [Stroke] explode all trees  18294  #6MeSH descriptor: [Hypertension] explode all trees 25793  #7 (cardiovascular disease): ti,ab,kw OR (disease cardiovascular): ti,ab,kw OR (cardiac events): ti,ab,kw OR (cardiac event): ti,ab,kw OR (event cardiac): ti,ab,kw OR (adverse cardiac event*): ti,ab,kw OR (cardiac event* adverse): ti,ab,kw OR (major adverse cardiac events): ti,ab,kwOR (heart attack): ti,ab,kw OR (CVD): ti,ab,kw OR (CHD): ti,ab,kw OR (MI): ti,ab,kw OR (HP): ti,ab,kw OR (IHD): ti,ab,kw 90262  #8 #1OR#2OR#3OR#4OR#5OR#6OR#7 216574  #9 (dietary inflammatory index): ti,ab, kw OR (DII): ti,ab, kw OR (inflammatory diet): ti,ab, kw OR (anti inflammatory diet): ti,ab, kw OR (dietary inflammation index): ti,ab, kw OR (anti inflammation diet): ti,ab, kw OR (inflammation diet): ti,ab, kw OR (dietary score): ti,ab, kw OR (diet scores): ti,ab, kw OR (Mediterranean diet): ti,ab, kw OR (DASH diet): ti,ab, kw OR(vegan diet ): ti,ab, kw OR (Nordic diet): ti,ab, kw OR (ketogenic diet): ti,ab, kw OR (vegetarian diet): ti,ab, kw OR (plant-based diet): ti,ab, kw 29616  #10 (random): ti,ab,kw OR (placebo): ti,ab,kw OR (double-blind): ti,ab,kw 1481144  #11 #8AND#9AND#10 4131 | ^25th^  Jan.  2025 | 4131 |
| CNKI | #1 major adverse cardiac events + cardiovascular disease + cardiovascular disease risk factors + cardiovascular disease (cvd) + cardiovascular disease risk + myocardial embolism + hypertension + heart disease + risk factors for cardiovascular disease + coronary heart disease + stroke + ischaemic heart disease 242,527  #2 Anti-Inflammatory Diet + Mediterranean Diet + Ketogenic Diet + DASH Diet + Desu Diet + Diet Inflammation Index + Inflammatory Diet + Diet Score + Vegan Diet + Traditional Nordic Diet + Anti-Inflammatory Diet + Plant-Based Diets 2081  #3 #1AND#2 338 | ^25th^  Jan.  2025 | 338 |

Supplementary table 6 retrieval formula
